# Suboptimal Selenium Intake Produces Sex-Specific Alterations in Metabolic Profiles in Western Diet-Fed Obese Mice

**DOI:** 10.3390/ijms27125345

**Published:** 2026-06-13

**Authors:** Sarah K. Walsh, Isabella Mezzani, Katy Pettigrew, John E. Hesketh, Giovanna Bermano

**Affiliations:** Centre for Obesity Research and Education (CORE), School of Pharmacy, Applied Sciences and Public Health, Robert Gordon University, Aberdeen AB10 7GJ, UK; s.walsh@rgu.ac.uk (S.K.W.); i.mezzani1@rgu.ac.uk (I.M.); j.hesketh@rgu.ac.uk (J.E.H.)

**Keywords:** selenium, metabolic syndrome, obesity, white adipose tissue, adipokines, leptin, sexual dimorphism

## Abstract

Selenium (Se) is an essential micronutrient required for redox regulation and metabolic homeostasis. Altered biomarkers of Se status have been linked with obesity and metabolic syndrome, yet its role in these conditions, particularly in a sex-specific context, is not well defined. This study investigated the impact of suboptimal Se intake on metabolic risk profiles in male and female mice with pre-existing diet-induced obesity. C57BL/6N mice were fed either a standard diet with adequate Se (SD-ASe), a Western diet with adequate Se (WD-ASe), or WD-ASe followed by a Western diet containing suboptimal Se levels (WD-SOSe). Metabolic parameters, adipokine profiles, tissue Se distribution, and gene expression in visceral white adipose tissue (vWAT) were assessed. Both sexes exhibited increased weight gain and adiposity in response to a Western diet; however, only males developed hypertension and elevated non-fasted blood glucose levels. Suboptimal Se intake elicited marked sex-dependent effects. In females, it induced elevated non-fasted blood glucose levels and circulating leptin, and further dysregulated circulating adipokine profiles, accompanied by pronounced alterations in selenoprotein expression and redox-related pathways in vWAT. In contrast, male mice exhibited a partial adaptation, including reduced glucose levels and minimal alterations in gene expression. Tissue Se distribution also appeared to be influenced by biological sex. These findings demonstrate that suboptimal Se intake may exacerbate obesity-related metabolic dysfunction in a sex-specific manner, with females showing greater susceptibility, underscoring the importance of micronutrient status and sex differences in metabolic disorders.

## 1. Introduction

The global rise in overweight and obesity is a major public health challenge, affecting nearly one-third of the population and contributing substantially to morbidity and mortality [1,2]. Obesity is a major risk factor for non-communicable diseases, including cardiovascular disease, type 2 diabetes mellitus, metabolic dysfunction-associated steatotic liver disease (MASLD), and several cancers. Central to these pathologies is the development of metabolic syndrome (MetS), characterised by insulin resistance, dyslipidaemia, hypertension, and impaired glucose homeostasis [3].

Obesity is marked by the expansion and dysfunction of adipose tissue, especially visceral white adipose tissue (vWAT), leading to chronic low-grade inflammation and oxidative stress driven by increased production of reactive oxygen species and pro-inflammatory mediators [4,5]. These alterations exacerbate metabolic dysfunction and increase the demand for micronutrients involved in antioxidant defence and redox regulation, while obesity-associated disruptions in micronutrient homeostasis may further impair metabolic health [6].

Selenium (Se) is an essential trace element that supports redox balance and metabolic function through its incorporation into selenoproteins, including glutathione peroxidases (GPXs), thioredoxin reductases (TRXRs), and iodothyronine deiodinases (DIOs) [7,8]. These proteins regulate antioxidant defence, thyroid hormone metabolism, and signalling pathways central to energy homeostasis [9,10]. Evidence increasingly links Se status to adipose tissue biology, including lipid metabolism, insulin sensitivity, and inflammatory responses (reviewed by Tinkov et al. [11]). However, epidemiological studies examining associations between Se status, obesity, and MetS have yielded inconsistent findings. Recent systematic reviews and meta-analyses suggest that associations between Se and metabolic health are context-dependent and influenced by factors such as biological sex, diet, and baseline metabolic status [12,13]. Emerging data further indicate sex-specific differences in Se metabolism and its metabolic consequences. For example, serum Se appears to exhibit an inverted U-shaped association with metabolic risk in males but a more linear relationship in females [14], potentially reflecting sex-dependent regulation of selenoprotein expression and tissue-specific prioritisation under limited Se availability [15].

In addition to micronutrient status, adipose tissue-derived hormones, particularly leptin, are key regulators of energy homeostasis and metabolic health. Beyond its central effects [16], leptin modulates lipid metabolism, insulin sensitivity, and inflammation, contributing to MetS [17]. Leptin is also closely linked to oxidative stress, a hallmark of adipose tissue dysfunction. Emerging evidence suggests a bidirectional interaction between Se status and leptin biology [18,19,20]. Through its role in antioxidant selenoproteins, Se may regulate leptin expression by modulating redox balance and inflammation in adipose tissue, while altered leptin levels may in turn affect Se distribution and metabolism. However, the interplay between leptin signalling and suboptimal Se intake, particularly under high-fat diet conditions and in a sex-specific context, remains unclear.

Previous studies have examined the roles of Se and individual selenoproteins in adipogenesis, lipid metabolism, oxidative stress [11,21,22], and observational data have linked Se status to obesity and MetS [12,13]. However, mechanistic insight into how reduced Se intake interacts with a high-fat diet to influence metabolic health remains limited, particularly in a sex-specific context. The effects of suboptimal Se status on lipid metabolism, redox balance, and inflammatory signalling under obesogenic conditions are not fully understood. To address this gap, this study investigated the impact of suboptimal Se intake in a mouse model of established diet-induced obesity. Male and female mice were rendered obese using a Western diet (WD) with adequate Se before being exposed to a diet with suboptimal Se levels, reflecting Se intakes reported in several European populations [23,24,25,26]. Parameters including glucose levels, lipid and adipokine profiles, and gene expression in vWAT were assessed to determine how Se intake modulates obesity-associated metabolic dysfunction in a sex-specific manner.

## 2. Results

### 2.1. Suboptimal Se Intake Differentially Affects Phenotype in Obese Male and Female Mice

**Figure 1:**
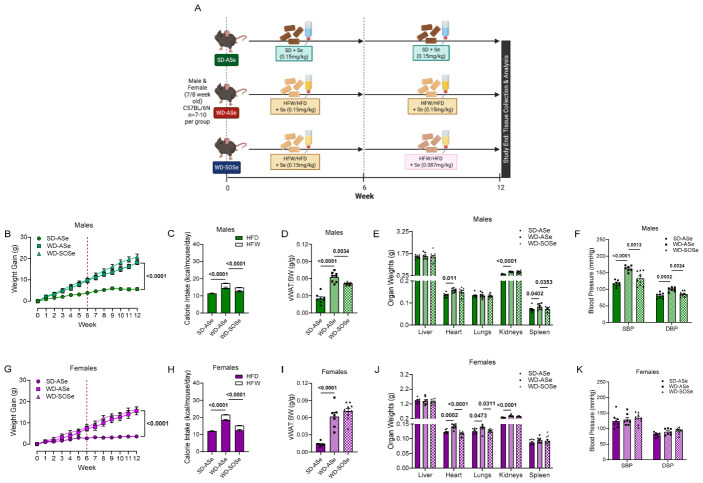
Suboptimal Se intake differentially affects the phenotype of obese male and female mice. Experimental design for dietary intervention study. Created in BioRender. Walsh, S. (2026) https://BioRender.com/pgruj76 (accessed on 1 April 2026) (**A**). Weight gain in male (**B**) and female (**G**) mice (red line indicates the commencement of the suboptimal Se dietary intervention in the WD-SOSe experimental groups). Average calorie intake in male (**C**) and female (**H**) mice. Ratio of vWAT to body weight (BW) in male (**D**) and female (**I**) mice. Tissue weights of male (**E**) and female (**J**) mice. Blood pressure (SBP and DBP) measurements in male (**F**) and female (**K**) mice. Weight gain between experimental groups was compared via a repeated measures ANOVA and Bonferroni post hoc test. Data from three experimental groups was analysed using a one-way ANOVA followed by Dunnett’s post hoc test. All data was expressed as the mean ± SEM, and results were considered statistically significant for *p* values ≤ 0.05. *n* = 7–10.

Both male and female mice fed a WD with adequate Se (WD-ASe) exhibited enhanced weight gain (both *p* < 0.001; Figure 1B,G) and increased vWAT accumulation (both *p* < 0.0001; Figure 1D,I) compared to mice fed a standard diet with adequate Se (SD-ASe). These findings were accompanied by an increased combined calorie intake (calories from high-fat diet (HFD) and high-fructose water (HFW)) in both groups of mice (both *p* < 0.0001; Figure 1C,H). Conversely, while weight gain (Figure 1B) remained elevated, both vWAT accumulation (*p* = 0.0034; Figure 1D) and calorie intake (*p* < 0.0001; Figure 1C) were decreased in male mice fed WD-ASe for 6 weeks followed by a WD containing suboptimal Se (WD-SOSe) for 6 weeks compared to those maintained on a WD-ASe throughout the entire dietary intervention period. Furthermore, despite a reduction in calorie consumption (*p* < 0.0001; Figure 1H), both vWAT accumulation (Figure 1I) and weight gain (Figure 1G) remained elevated in female WD-SOSe mice compared to their counterparts in the WD-ASe group. In relation to other organs, while liver weight was not altered in male mice in response to any of the diets used, both heart and kidney weights were significantly increased in response to the WD (*p* = 0.011 and *p* < 0.0001, respectively; Figure 1E), and these effects were not influenced by the Se content of the diet. Furthermore, spleen weight was increased in the male WD-ASe group compared to the equivalent SD-ASe group (*p* = 0.0402; Figure 1E), and this effect was reversed in response to suboptimal Se intake (*p* = 0.0353; Figure 1E). In female mice, both heart and kidney weights increased in response to a WD with adequate Se (*p* = 0.0002 and *p* < 0.0001, respectively; Figure 1J). In contrast, suboptimal Se intake reduced heart weight in WD-fed mice (*p* < 0.0001), while kidney weight remained elevated (Figure 1J). Neither liver nor spleen weight was affected by any of the diets used in female mice (Figure 1J). Male mice in the WD-ASe group developed hypertension, characterised by significant increases in both systolic blood pressure (SBP) and diastolic blood pressure (DBP) compared with mice on a standard diet (SD) (*p* < 0.0001 and *p* = 0.0002, respectively; Figure 1F). In contrast, male mice fed a WD with suboptimal Se exhibited significantly lower SBP and DBP compared to those in the WD-ASe group (*p* = 0.0013 and *p* = 0.0024, respectively; Figure 1F). Finally, neither SBP nor DBP was significantly affected by any of the diets used in female mice (Figure 1K).

### 2.2. Suboptimal Se Intake Increases Non-Fasted Blood Glucose Levels and Worsens Hyperleptinemia in Female Mice

**Figure 2:**
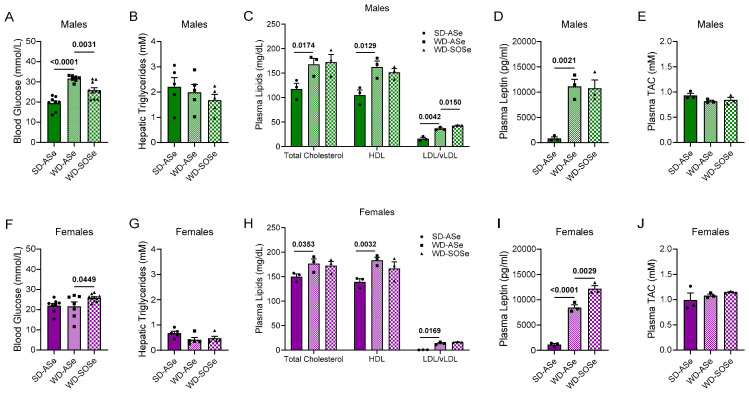
Suboptimal Se intake increases non-fasted blood glucose levels and worsens hyperleptinemia in female mice. Non-fasted blood glucose in male (**A**) and female (**F**) mice. Hepatic triglycerides in male (**B**) and female (**G**) mice. Circulating lipids in male (**C**) and female (**H**) mice. Plasma leptin in male (**D**) and female (**I**) mice. TAC in the plasma of male (**E**) and female (**J**) mice. For plasma lipid, leptin and TAC measurements, three separate pooled plasma samples were prepared for each experimental group by combining the plasma from different combinations of 3–5 mice from that group, and then three independent experiments were conducted. Data from the three experimental groups was analysed using a one-way ANOVA followed by Dunnett’s post hoc test. All data was expressed as the mean ± SEM, and results were considered statistically significant for *p* values ≤ 0.05. *n* = 3–10.

Non-fasted blood glucose concentration was significantly increased in male (*p* < 0.0001; Figure 2A), but not female (Figure 2F) mice in the WD-ASe experimental group compared to control mice on a SD-ASe. Conversely, in response to a WD that contained suboptimal Se, circulating glucose levels were reduced in male mice (*p* = 0.0031; Figure 2A), while they were significantly increased in female mice (*p* = 0.0449; Figure 2F). Measurement of hepatic triglycerides revealed that although the latter were more abundant in the livers of male (0.97–3.06 mM; Figure 2B) compared to female mice (0.25–0.91 mM; Figure 2G), they were not significantly altered by any of the dietary interventions used in the present study. However, in terms of circulating lipids, total cholesterol, high-density lipoproteins (HDL), and low-density lipoproteins/very low-density lipoproteins (LDL/vLDL) were all significantly increased in the plasma of both male (*p* = 0.0174, *p* = 0.0129 and *p* = 0.0042, respectively; Figure 2C) and female (*p* = 0.0353, *p* = 0.0032 and *p* = 0.0169, respectively; Figure 2H) mice in response to a WD with adequate Se. Furthermore, while the reduction of Se in the WD diet did not alter circulating lipids in female mice (Figure 1H), the concentration of LDL/vLDL was significantly increased in the plasma of male mice in the WD-SOSe group compared to the WD-ASe group (*p* = 0.0150; Figure 2C). In response to the WD-induced increased adiposity, leptin concentration was increased in the plasma of both male (*p* = 0.0021; Figure 2D) and female (*p* < 0.0001; Figure 2I) mice fed a WD with adequate Se. However, while this hyperleptinemia was not altered in response to suboptimal Se intake in male mice (Figure 2D), it was significantly enhanced in female mice in the WD-SOSe experimental group (*p* = 0.0029; Figure 2I). Finally, given the role of Se as an antioxidant, total antioxidant capacity (TAC) was measured in the plasma of all mice; however, this did not differ significantly in response to any of the diets used in either sex (Figure 2E,J).

### 2.3. WD and Suboptimal Se Dysregulate Circulating Adipokine Profiles

**Figure 3:**
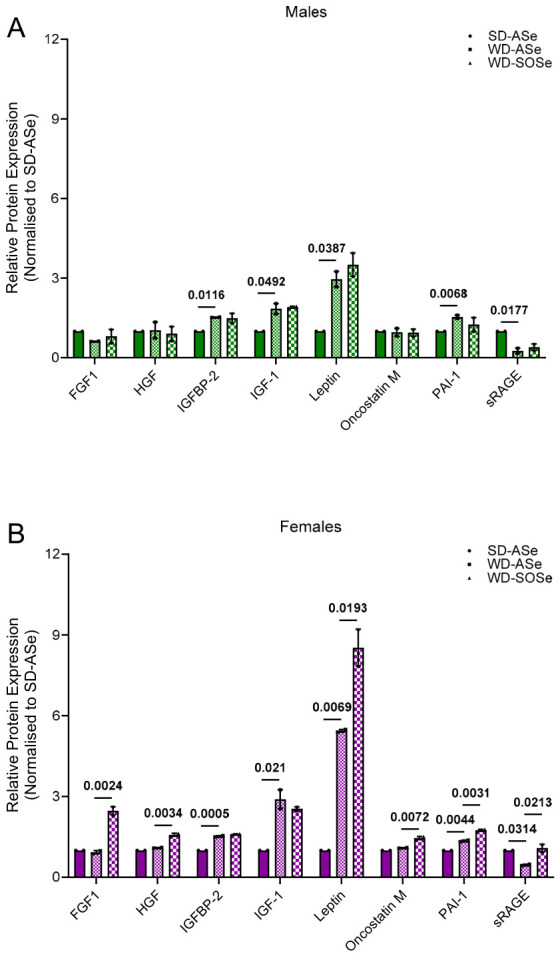
WD and suboptimal Se dysregulate circulating adipokine profiles. Circulating adipokines were measured in pooled plasma from both male (**A**) and female (**B**) mice using the Proteome Profiler Mouse Adipokine Array Kit. Two separate pooled plasma samples were prepared for each experimental group by combining the plasma from different combinations of 3–5 mice from that group, and then two independent experiments were conducted. Data from three experimental groups was analysed using a one-way ANOVA followed by Dunnett’s post hoc test. All data was expressed as the mean ± SEM, and results were considered statistically significant for *p* values ≤ 0.05. *n* = 2.

Preliminary data from a protein array revealed that, while 38 adipokines were detected in the plasma of both male and female mice (see Supplemental Tables S1 and S2), the relative concentrations of only a selected number of adipokines were increased in response to a WD (Figure 3). In male mice in the WD-ASe group, the relative concentrations of the following adipokines: insulin-like growth factor-binding protein 2 (IGFBP-2; 1.52-fold, *p* = 0.0116), insulin-like growth factor 1 (IGF-1; 1.85-fold, *p* = 0.0492), leptin (2.96-fold, *p* = 0.0387), and plasminogen activator inhibitor-1 (PAI-1; 1.54-fold, *p* = 0.0068) were all increased, while the concentration of soluble receptor for advanced glycation end-products (sRAGE; 0.26-fold, *p* = 0.0177) was decreased in the plasma compared to mice on a SD (Figure 3A). Furthermore, varying the Se content of the WD administered to male mice did not alter the profile of the adipokines detected (Figure 3A). Conversely, female mice appeared to be more susceptible to variations in Se content, as the secretion of several additional adipokines was enhanced in response to suboptimal Se (Figure 3B). Similar to male mice, the relative concentrations of the following adipokines: IGFBP-2 (1.52-fold, *p* = 0.0024), IGF-1 (2.90-fold, *p* = 0.021), leptin (5.44-fold, *p* = 0.0069), and PAI-1 (1.35-fold, *p* = 0.0044) were all increased, while the concentration of sRAGE (0.46-fold, *p* = 0.0314) was decreased in the plasma of female mice in the WD-ASe group (Figure 3B). Finally, a reduction of Se content in the WD induced the secretion of the following additional adipokines: fibroblast growth factor 1 (FGF1; 2.47-fold, *p* = 0.0024), hepatocyte growth factor (HGF; 1.58-fold, *p* = 0.0034), and oncostatin M (1.46-fold, *p* = 0.0072) in female mice. This also enhanced the secretion of leptin (8.53-fold, *p* = 0.0193) and PAI-1 (1.75-fold, *p* = 0.0031), and restored plasma levels of sRAGE (1.08-fold, *p* = 0.0213) compared to the WD-ASe experimental group (Figure 3B).

### 2.4. Biological Sex Influences Both the Preferential Storage of Se Within Different Tissues and the Response to Alterations in Se Intake

**Figure 4:**
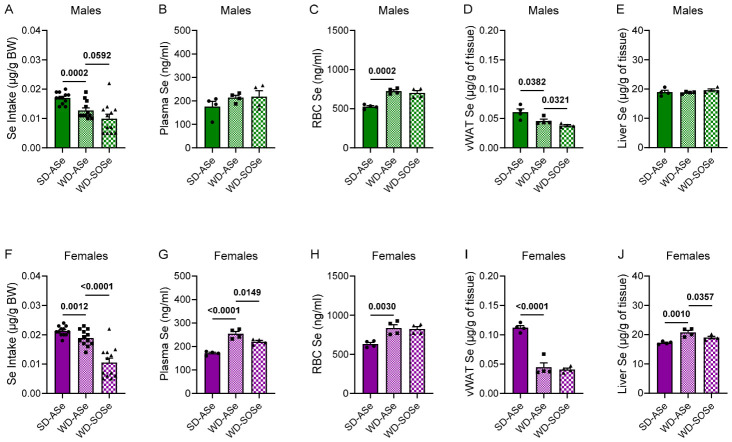
Biological sex influences both the preferential storage of Se within different tissues and the response to alterations in Se intake. Se intake in male (**A**) and female (**F**) mice (*n* = 8–10). Plasma Se in male (**B**) and female (**G**) mice. RBC Se content in male (**C**) and female (**H**) mice. vWAT Se content in male (**D**) and female (**I**) mice. Liver Se content in male (**E**) and female (**J**) mice. Data from three experimental groups was analysed using a one-way ANOVA followed by Dunnett’s post hoc test. All data was expressed as the mean ± SEM, and results were considered statistically significant for *p* values ≤ 0.05. *n* = 4.

To control calorific intake, food consumption was decreased in all mice fed a WD compared to those on a SD. Consequently, Se intake was significantly reduced in both male (*p* = 0.0002; Figure 4A) and female (*p* = 0.0012; Figure 4F) mice fed a WD-ASe, and the intake of this trace element was reduced further in male (*p* = 0.0592; Figure 4A) and female (*p* < 0.0001; Figure 4F) mice fed a WD containing suboptimal Se. However, despite reductions in Se intake in WD-fed male mice, neither the plasma (Figure 4B) nor the hepatic (Figure 4E) concentration of Se was significantly altered in these animals, while, surprisingly, the Se content within red blood cells (RBC) was significantly increased (*p* = 0.0002; Figure 4C). Conversely, Se accumulation within the vWAT of male mice fed a WD with adequate Se was significantly decreased compared to those on a SD (*p* = 0.0382; Figure 4D) and was reduced further in those mice given a WD with suboptimal Se (*p* = 0.0321; Figure 4D). Female mice fed a WD with adequate Se responded to a reduction in Se intake (i.e., compared to those on a SD) with increases in both circulating (plasma (*p* < 0.0001; Figure 4G) and RBC (*p* = 0.0030; Figure 4H)) and hepatic (*p* = 0.0010; Figure 4J) Se. Furthermore, Se concentration in both plasma (*p* = 0.0149; Figure 4G) and liver (*p* = 0.0357; Figure 4J) was significantly reduced in female mice from the WD-SOSe experimental group, while RBC Se content (Figure 4H) remained unchanged compared to those animals in the WD-ASe group. In female mice fed a SD, Se accumulation in vWAT was almost double that observed in male mice on the equivalent diet (0.12 ± 0.004 vs. 0.061 ± 0.006 μg/g of tissue; Figure 4D,I). However, like male mice, vWAT Se content was decreased in female mice from the WD-ASe group compared to those on the SD (*p* < 0.0001; Figure 4I), but, unlike males, was not decreased further in response to the WD with suboptimal Se (Figure 4I).

### 2.5. Alterations in Both Dietary Fat and Se Content Influence the Expression of Selenoproteins and Markers of Both Redox Status and Energy Metabolism in the vWAT of Female Mice

**Figure 5:**
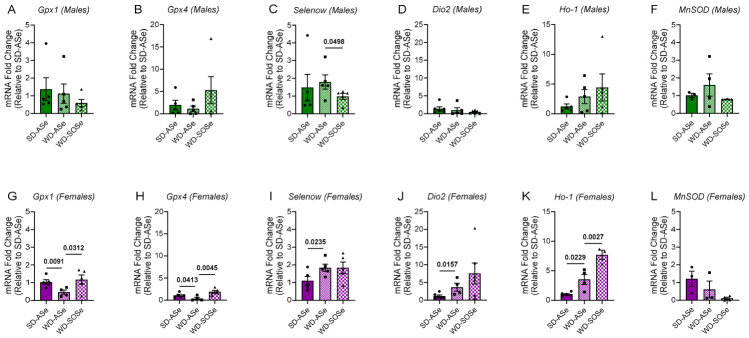
Alterations in both dietary fat and Se content influence the expression of markers of redox status and selenoproteins in the vWAT of female mice. Gene expression was normalised to the SD-ASe experimental group. Relative expression of *Gpx1* (**A**), *Gpx4* (**B**), *Selenow* (**C**), *Dio2* (**D**), *Ho-1* (**E**), and *MnSOD* (**F**) in the vWAT of male mice. Relative expression of *Gpx1* (**G**), *Gpx4* (**H**), *Selenow* (**I**), *Dio2* (**J**), *Ho-1* (**K**), and *MnSOD* (**L**) in the vWAT of female mice. Data from the three experimental groups was analysed using a one-way ANOVA followed by Dunnett’s post hoc test. All data was expressed as the mean ± SEM, and results were considered statistically significant for *p* values ≤ 0.05. *n* = 3–6.

The impact of both a WD and suboptimal Se intake on the mRNA expression of selected selenoproteins (glutathione peroxidase 1 (*Gpx1*), glutathione peroxidase 4 (*Gpx4*), selenoprotein W (*Selenow*), and type II iodothyronine deiodinase (*Dio2*)) and markers of redox status (heme oxygenase 1 (*Ho-1*) and manganese superoxide dismutase (*MnSOD*)) was investigated in the vWAT of all mice. In male mice, the expression of *Gpx1*, *Gpx4*, *Dio2*, *Ho-1*, and *MnSOD* did not differ significantly in response to any of the dietary interventions tested (Figure 5A–F), while the expression of the selenoprotein, *Selenow*, was significantly reduced by approximately 50% in response to suboptimal Se in WD-fed mice (*p* = 0.0498; Figure 5C). In contrast, significant alterations in the expression of both selenoproteins and markers of redox stress were observed in the vWAT of female mice in response to both formulations of the WD. In particular, the expression of *Gpx1* (0.46-fold, *p* = 0.0091; Figure 5G) and *Gpx4* (0.37-fold, *p* = 0.0413; Figure 5H) were decreased, whereas the expression of *Selenow* (1.84-fold, *p* = 0.0235; Figure 5I), *Dio2* (3.84-fold, *p* = 0.0157; Figure 5J), and *Ho-1* (3.54-fold, *p* = 0.0229; Figure 5K) were all increased in response to a WD with adequate Se. The expression of *MnSOD* (Figure 5L) remained unchanged compared to SD-ASe. In female mice fed a WD containing suboptimal Se, the expression of *Gpx1* (1.17-fold, *p* = 0.0312; Figure 5G), *Gpx4* (1.89-fold, *p* = 0.0045; Figure 5H), and *Ho-1* (7.69-fold, *p* = 0.0027; Figure 5K) in the vWAT were all significantly increased compared to those mice in the WD-ASe experimental group. Furthermore, while the expression of *Selenow* and *Dio2* were not altered in response to suboptimal Se, the expression of *MnSOD* was reduced by approximately 80% (Figure 5L) in the vWAT of female mice in the WD-SOSe experimental group.

**Figure 6:**
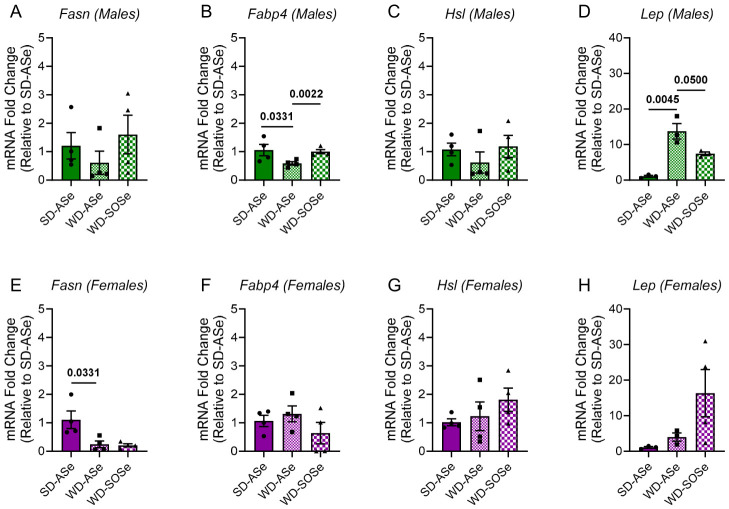
Alterations in both dietary fat and Se content influence the expression of markers of lipid and energy metabolism in the vWAT of male mice. Gene expression was normalised to the SD-ASe experimental group. Relative expression of *Fasn* (**A**), *Fabp4* (**B**), *Hsl* (**C**), and *Lep* (**D**) in the vWAT of male mice. Relative expression of *Fasn* (**E**), *Fabp4* (**F**), *Hsl* (**G**), and *Lep* (**H**) in the vWAT of female mice. Data from three experimental groups was analysed using a one-way ANOVA followed by Dunnett’s post hoc test. All data was expressed as the mean ± SEM, and results were considered statistically significant for *p* values ≤ 0.05. *n* = 3–4.

The effect of the different dietary interventions on the expression of genes relating to adipocyte differentiation, lipid, and energy metabolism (i.e., fatty acid synthase (*Fasn*), fatty acid binding protein 4 (*Fabp4*), hormone-sensitive lipase (*Hsl*), and leptin (*Lep*)) in the vWAT was investigated. In response to a WD with adequate Se, the expression of *Fasn* (Figure 6A) and *Hsl* (Figure 6C) remained unchanged, while the expression of *Fabp4* (0.59-fold, *p* = 0.0331; Figure 6B) was decreased, and the expression of *Lep* (14.35-fold, *p* = 0.0045; Figure 6D) increased in the vWAT of male mice. Conversely, the expression of *Fabp4* (Figure 6B) was restored to control levels (i.e., similar to that observed in the vWAT of mice on a SD) and the expression of *Lep* (7.35-fold, *p* = 0.0500; Figure 6D) significantly reduced in the vWAT of male mice fed a WD containing suboptimal Se. In the vWAT of female mice, the gene expression of *Fabp4*, *Hsl*, and *Lep* all remained unchanged in response to a WD with adequate Se, while the expression of *Fasn* (0.25-fold, *p* = 0.0331; Figure 6E) was significantly reduced compared to mice on a SD. In contrast to male mice, the gene expression of the following markers relating to adipocyte differentiation, lipid, and energy metabolism (i.e., *Fasn*, *Fabp4*, *Hsl*, and *Lep*) remained unchanged in the vWAT of female mice in the WD-SOSe experimental group compared to those in the WD-ASe group (Figure 6E–H).

## 3. Discussion

The main aim of this study was to examine the combined impact of both suboptimal Se intake and biological sex on MetS risk factors in the setting of pre-existing obesity. In previously published studies, male mice have been shown to exhibit more exaggerated responses in terms of their metabolic risk factor profile when administered an obesity-inducing diet (i.e., a WD) [27,28,29,30,31,32,33]. This study confirmed these previous findings, as although both male and female mice were characterised by increased weight gain, enhanced visceral adiposity, dyslipidaemia, hyperleptinaemia, and dysregulated circulating adipokine profiles, only male mice exhibited both elevated non-fasted blood glucose and hypertension in response to a WD. Conversely, when both groups of mice were exposed to a WD containing suboptimal Se, improvements in some metabolic risk factors (i.e., reductions in visceral adiposity, blood glucose and blood pressure) were observed in male mice, while detrimental changes in several of the same parameters (i.e., maintained visceral adiposity, elevated blood glucose and enhanced hyperleptinaemia) were observed in female mice, illustrating the influence of biological sex.

Given that suboptimal Se status is typically linked to exacerbated obesity, hyperglycaemia, and hypertension [34,35,36,37], it seems unlikely that reduced Se intake in male mice is responsible for their improved metabolic risk factor profile, although this possibility cannot be completely excluded. An alternative explanation for the reductions in blood glucose, visceral adiposity and blood pressure in this experimental group may involve, at least in part, the reduced caloric intake observed in these animals. Both male and female mice in the suboptimal Se experimental groups consumed fewer calories (due to reductions in both food and fructose water intake) than their WD-ASe counterparts, which was unexpected as several studies have demonstrated that food intake in mice is increased when the Se content of the chow is decreased [34,37]. Given the well-established benefits of calorie restriction (CR) in mice, including reduced adiposity, improved hyperglycaemia, and attenuated hypertension [38,39,40,41,42,43,44,45], it is plausible that the favourable modulation of metabolic risk factor markers observed in WD-SOSe male mice are driven, at least in part, by reduced caloric intake, potentially masking detrimental effects or amplifying any beneficial effects of suboptimal Se intake.

In contrast, despite also consuming fewer calories than their WD-ASe counterparts, female mice in the WD-SOSe experimental group exhibited a worse metabolic risk factor profile, which may suggest that the beneficial effects of CR are lacking and/or blunted in female mice. This observation is consistent with previous studies that demonstrated the positive effects of CR are more prominent in male rodents [46,47,48]. The impact of suboptimal Se status also appears to be influenced by biological sex, as lower circulating Se levels correlate with obesity and diabetes in females, but not in males [23,26,49]. In contrast, evidence for a comparable sex-specific effect of suboptimal Se status on metabolic risk factor profiles in rodents remains limited, as most preclinical studies use a single sex [37,50,51,52] and/or focus on Se supplementation in models where control animals already receive Se-adequate diets [34,53,54,55,56]. Nevertheless, the few studies incorporating both sexes and Se-deficient control groups report both the absence [57] and the presence [35] of sex-specific differences in the way in which suboptimal Se status influences metabolic outcomes in mice. Consistent with our findings, neither of the aforementioned studies reported significant changes in fat mass in HFD-fed female mice in response to suboptimal Se intake [35,57]. Furthermore, female WD-SOSe mice exhibited exacerbated hyperleptinaemia, consistent with reports of elevated circulating leptin in mice fed a HFD containing suboptimal Se [34,35].

Concomitant with the increased adiposity observed in both male and female WD-fed mice, preliminary data from our study indicates dysregulation of circulating adipokine profiles, which aligns with previous reports in rodent diet-induced obesity models [34,58,59,60]. However, unlike their male counterparts, the circulating adipokine profile of female WD-SOSe mice appeared more dysregulated and was also characterised by changes in FGF1, HGF, leptin, oncostatin M, PAI-1, and sRAGE, highlighting a sex-specific effect of suboptimal Se intake that has not previously been reported. The overall impact of the changes in the adipokine profile observed in female WD-SOSe mice on their metabolic risk factor profile is challenging to infer as several of the affected adipokines have opposing roles. FGF1 [61,62], HGF [63], and sRAGE [64] are associated with protective or compensatory actions, including improved glucose tolerance, whereas oncostatin M [65,66] and PAI-1 [67] drive inflammatory signalling and glucose intolerance. As female WD-SOSe mice in the present study exhibited elevated non-fasted blood glucose levels, it is possible that this dysregulated adipokine profile may contribute to their worsened metabolic phenotype; however, this requires full investigation and validation.

While the mechanisms underpinning the influence of biological sex on organ-specific Se distribution have yet to be elucidated, studies have shown that Se absorption is increased in both women and female rodents and that the latter are better at maintaining circulating Se levels compared to male rodents (reviewed by Seale et al. [68]). In the present study, diet- and/or sex-dependent differences in organ-specific Se distribution may contribute to the phenotypic and biochemical alterations observed in these mice. Although not identical to the present findings, other mouse studies have similarly support hierarchical Se allocation across tissues, with diet- and stress-dependent modulation of Se distribution [34,69,70,71,72]. Under Se-adequate WD conditions, Se was redistributed to plasma and RBC, consistent with systemic glutathione peroxidase-dependent redox control. This redistribution was more pronounced in female mice, which aligns with the previously reported estradiol-induced increases in both plasma and RBC Se content [73], whereas male mice maintained stable plasma Se, indicative of tighter homeostatic regulation [74,75,76]. The liver remained Se-replete in males despite reduced intake, while females showed increased hepatic Se, consistent with greater adaptive plasticity [77,78,79]. By contrast, Se was depleted from vWAT in both sexes, particularly females, supporting its role as a lower-priority reserve under metabolic stress [56,77,80].

Under suboptimal Se intake, these adaptive responses were constrained, particularly in female mice, as reflected by lower hepatic and plasma Se. This is consistent with earlier studies showing that hepatic Se declines once intake is insufficient to sustain selenoprotein synthesis and systemic distribution [68,70,71,77,78,79]. Together, these findings suggest that WD-induced metabolic stress promotes Se redistribution from storage to functional compartments, with a stronger adaptive response in female mice. This contrasts with the preferential hepatic and adipose Se accumulation reported under supra-optimal Se intake in WD models [54].

The depletion of Se in vWAT tissue may contribute to the altered metabolic risk factor profiles of these mice by modulating, in part, adipose tissue gene expression profiles. In Se-adequate WD-fed female mice, reduced *Gpx1* and *Gpx4* alongside increased *Selenow*, *Dio2* and *Ho-1* expression suggest adaptive reconfiguration of the selenoprotein hierarchy, consistent with redox and metabolic remodelling [15,81,82,83,84]. This profile aligns with greater adipose plasticity and stress-responsive signalling in females under obesogenic conditions [82,83,84]. Furthermore, the combination of both reduced expression of *Gpx1* and *Gpx4* and increased expression of *Ho-1* indicates changes in redox status within vWAT, which is known to contribute to a worse metabolic profile [85,86]. Induction of *Dio2* has been shown to activate metabolic pathways increasing energy expenditure and consequently reducing fat mass; however, this effect appears to be dependent on adipose tissue type (e.g., brown adipose tissue, subcutaneous WAT) and is absent in visceral/epididymal WAT [87,88]. By contrast, male mice showed minimal transcriptional changes, consistent with lower adipose plasticity. Under suboptimal Se intake, female mice showed a compensatory antioxidant response, with increased *Gpx1*, *Gpx4* and *Ho-1* expression, which may suggest heightened oxidative stress under WD-induced lipid overload. Despite reduced Se intake, hierarchical control appears to prioritise essential selenoproteins—particularly GPX4—thereby limiting lipid peroxidation [77,89], whereas male mice again showed a constrained response largely restricted to *Selenow*.

Building on the diet- and sex-dependent redistribution of Se and changes in adipose gene expression described above, WD and Se intake may further interact to shape adipocyte lipid metabolism and endocrine function in a sex-specific manner. In male mice, Se-adequate WD increased *Lep* and reduced *Fabp4* expression relative to SD-ASe controls, consistent with altered adipocyte endocrine signalling and lipid handling [90,91]. In female mice, Se-adequate WD reduced the expression of *Fasn*, which may indicate a shift away from de novo lipogenesis [92,93,94]. Suboptimal Se intake disrupted this adaptive profile in male mice, with reduced *Lep* and increased *Fabp4* expression, whereas female mice maintained adipokine expression alongside increased antioxidant gene expression, consistent with stronger redox buffering capacity. Together, these data indicate that suboptimal Se intake differentially remodels vWAT pathways in obese male and female mice fed a WD. In female mice, Se availability was associated with coordinated changes in selenoproteins, redox-related genes and lipid-handling markers, supporting metabolic flexibility under Se sufficiency and enhanced antioxidant activity under limiting Se intake. By contrast, male mice showed a more restricted transcriptional response, with suboptimal Se intake preferentially disrupting FABP4–leptin-associated signalling.

In conclusion, suboptimal Se intake in the context of diet-induced obesity may drive sex-dependent changes in metabolic risk factor profile, with female mice appearing to show greater susceptibility to adverse alterations compared to male mice. Specifically, suboptimal Se intake in females was associated with elevated non-fasted blood glucose levels, a shift in adipokine profile and altered expression of genes regulating redox status, whereas males showed more modest and, in the case of some parameters, potentially adaptive responses. These findings suggest that micronutrient intake, obesity, and/or biological sex may interact to influence metabolic regulation, and support the importance of considering micronutrient status as a potentially modifiable factor in obesity-related metabolic disease, with possible implications for sex-specific nutritional approaches in research and clinical practice.

This study has several strengths, including the use of a clinically relevant model of obesity combined with suboptimal Se intake, reflecting nutritional patterns observed in European populations [24,95]. Moreover, the inclusion of both sexes provides important insight into biological variability that is often overlooked in preclinical research. However, some limitations should be acknowledged. The study did not directly assess insulin sensitivity, which would have strengthened conclusions regarding metabolic dysfunction, and links between Se intake, adipokine profiles and redox-related gene expression will require further study, including protein-level validation.

## 4. Materials and Methods

### 4.1. Animal Housing and Ethical Approval

The animal studies were conducted under an appropriate Project License in accordance with the UK Animals (Scientific Procedures) Act 1986. Male and female C57Bl/6N mice (7–8 weeks old) were purchased from Charles River (Kent, UK) and housed within the Biological Services Unit (BSU) at Robert Gordon University in a temperature- and humidity-controlled room (19–23 °C and 45–65% respectively) set on a 12 h light/dark cycle (7 a.m.–7 p.m.), and the mice were maintained in accordance with the husbandry guidelines set by the UK Home Office. Prior to the commencement of any experiments, the mice were allocated 7 days for acclimatisation within the BSU, with all dietary interventions and surgical procedures conducted within the BSU. All in vivo work is reported in accordance with the ARRIVE guidelines [96].

### 4.2. Dietary Intervention

Male and female C57Bl/6N mice (7–8 weeks old) were randomly assigned to one of three dietary interventions (see Figure 1A for experimental design): (1) SD-ASe: standard diet containing 15 kcal% fat with 0.15 mg/kg Se (standard chow deficient in Se (C1045, Altromin, Lage, Germany) supplemented with selenomethionine to 0.15 mg/kg Se) and regular drinking water for 12 weeks; (2) WD-ASe: high-fat diet (HFD) containing 60 kcal% fat with 0.15 mg/kg Se (Se content of high fat chow (C1088-02, Altromin, Lage, Germany) adjusted to 0.15 mg/kg Se via the addition of selenomethionine) for 12 weeks and 30% (*w*/*v*) fructose in drinking water (HFW) for 11 weeks (i.e., Western Diet (WD)); and (3) WD-SOSe: HFD containing 60 kcal% fat with 0.15 mg/kg Se for 6 weeks followed by HFD containing 60 kcal% fat with 0.087 mg/kg Se (Se content of high fat chow (C1088-02, Altromin, Lage, Germany) adjusted to 0.087 mg/kg Se via the addition of selenomethionine) for a further 6 weeks and 30% (*w*/*v*) fructose in drinking water for 11 weeks. Food intake and body weights were recorded weekly.

### 4.3. Blood Pressure Measurements in Mice

The mice were weighed and anaesthetised with a mixture of ketamine (120 mg/kg; Narketan, Vetoquinol UK Ltd., Northamptonshire, UK) and xylazine (16 mg/kg; Rompun^®^, Bayer, Dublin, Ireland) via intraperitoneal injection. Blood pressure (systolic (SBP) and diastolic (DBP) blood pressure) was measured via the insertion of a 1.4-Fr pressure conductance catheter (SPR-839; Millar Inc., Houston, TX, USA) into the right carotid artery. Following the measurement of arterial pressure, whole blood was collected via cardiac puncture into a heparinised tube (final heparin concentration of 20 U/mL of blood) for further analysis.

### 4.4. Biochemical Analysis in Blood and Plasma

Blood glucose was measured in samples from individual mice using a glucometer (CodeFree™ Glucometer, SD Biosensor, Poway, CA, USA) and the remaining blood sample centrifuged at 2500× *g* for 15 min at 4 °C to obtain plasma and red blood cells (RBC). Circulating levels of total cholesterol, HDL, and LDL/vLDL concentrations were all measured using the Cholesterol Assay Kit (cat# ab65390; Abcam, Cambridge, UK) as per the manufacturer’s instructions. TAC and leptin concentration were measured in plasma using the Antioxidant Assay Kit (cat# 709001; Cayman Chemical, Ann Arbor, MI, USA) and the Mouse Duoset Leptin ELISA (cat# DY498; Biotechne, Minneapolis, MN, USA), respectively, as per the manufacturer’s instructions. Circulating levels of adipokines were measured in plasma using the Proteome Profiler Mouse Adipokine Array Kit (cat# ARY013; Biotechne, Minneapolis, MN, USA) as per the manufacturer’s instructions.

### 4.5. Measurement of Hepatic Triglycerides

Liver tissue samples (*n* = 5 per experimental group) were homogenised in a 5% Nonidet P-40 solution (*v*/*v* in water) at a ratio of 10 mg/100 μL. The homogenised samples were slowly heated to 90 °C over 5 min, and this step repeated. Following centrifugation at 13,000× *g* for 3 min, the supernatant was removed, diluted 1:10 with water, and triglycerides quantified using the Triglyceride Assay Kit (cat# ab65336; Abcam, Cambridge, UK) as per the manufacturer’s instructions.

### 4.6. Selenium Measurement

Selenium content in plasma, red blood cells (RBC), liver, and visceral white adipose tissue (vWAT) was measured via inductively coupled plasma mass spectrometry (ICP-MS) (Agilent 7700x ICPMS with MassHunter 4.6 Workstation, Agilent Technologies, Santa Clara, CA, USA) after digestion with nitric acid and hydrogen peroxide. Pooled samples (*n* = 5 mice per sample) were analysed from each experimental group in four independent analyses and comprised either 250 mg of tissue (50 mg per mouse) or 125 μL of plasma/RBC (25 μL per mouse).

### 4.7. Gene Expression

RNA was extracted from vWAT (approximately 100 mg) using TRI^®^ Reagent Solution (Invitrogen, Paisley, UK) following the manufacturer’s guidelines. RNA concentration and purity were measured using UV absorbance spectrophotometry, with an adequate sample ratio A260/A280 being ≥1.8. Three to six separate pooled cDNA samples were prepared for each experimental group by combining the RNA from different combinations of 3–5 mice from that group. For the preparation of cDNA, 1 µg of pooled RNA was reverse-transcribed using the High-Capacity cDNA Reverse Transcription Kit (Applied Biosystems, Warrington, UK) according to the manufacturer’s instructions. Briefly, each reaction consisted of 2 µL of 10X RT Buffer, 0.8 µL of 25X dNTP Mix, 2 µL of 10X Random Primers, 1 µL of MultiScribe™ Reverse Transcriptase, 3.2 µL DNase/RNase free water, and 1 µg of RNA, in a final volume of 20 µL. Reactions were performed on the T3000 Thermocycler (Biometra, Göttingen, Germany) as follows: 25 °C for 10 min, 37 °C for 120 min, 85 °C for 5 min, and then cooled to 4 °C. The cDNA was diluted with DNase/RNase free water to obtain the equivalent of 50 ng/µL of starting RNA and stored at −20 °C. qPCR was performed with SYBR Green PCR Master Mix (Primer Design, Manchester, UK) on a 7900 HT Fast Real-Time PCR system (Applied Biosystems, Warrington, UK) as follows: an initial hot start at 95 °C for 15 min, followed by 40 cycles of 95 °C for 30 s and annealing at 60 °C for 2 min. Beta-2 microglobulin (*B2m*) was amplified as an internal control. Primers were designed using Pubmed (Entrez Gene), and the NCBI primer blast tool (National Center for Biotechnology Information (NCBI), Bethseda, MD, USA) https://www.ncbi.nlm.nih.gov/tools/primer-blast/, accessed on 19 January 2020. Sequences are reported in Table 1. mRNA levels were quantified using the comparative CT method (2^−∆∆Ct^ method) with the data normalised to *B2m* gene expression. Three to six independent qPCR experiments were conducted comparing all experimental groups.

### 4.8. Statistical Analysis

Statistical analysis was performed using GraphPad Prism 10 software (GraphPad Software, La Jolla, CA, USA) and the specific statistical tests used detailed in each of the figure legends. All data was expressed as the mean ± SEM, and results were considered statistically significant for *p* values ≤ 0.05.

## Figures and Tables

**Table 1:** Sequence of mouse primers used for qPCR.

Gene	Forward Primer	Reverse Primer	Product Size (bp)
*B2m*	TGGTCTTTCTGGTGCTTGTCT	GGATTTCAATGTGAGGCGGG	153
*Fabp4*	ATGATCATCAGCGTAAATGG	GCCTTTCATAACACATTCCA	242
*Fasn*	AAGCGGTCTGGAAAGCTGAA	AGGCTGGGTTGATACCTCCA	150
*Dio2*	ATGGGACTCCTCAGCGTAGAC	ACTCTCCGCGAGTGGACTT	150
*Gpx1*	CAGGAGAATGGCAAGAATGA	GAAGGTAAAGAGCGGGTGAG	135
*Gpx4* [97]	GCTGGGAAATGCCATCAAATGG	ACGGCAGGTCCTTCTCTATCAC	115
*Ho-1*	CGCTACCTGGGTGACCTCTC	TGTTTGAACTTGGTGGGGCT	134
*Hsl*	GATTTACGCACGATGACACAGT	ACCTGCAAAGACATTAGACAGC	113
*Lep*	GTTCCTGTGGCTTTGGTCCT	ATACCGACTGCGTGTGTGAAA	130
*MnSod*	GCCTGCTCTAATCAGGACC	GTAGTAAGCGTGCTCCCACA	84
*Selenow* [97]	ATGCCTGGACATTTGTGGCGA	GCAGCTTTGATGGCGGTCAC	153

*B2m:* beta 2 microglobulin; *Fabp4*: fatty acid-binding protein 4; *Fasn*: fatty acid synthase; *Dio2*: type II iodothyronine deiodinase; *Gpx1*: glutathione peroxidase 1; *Gpx4*: glutathione peroxidase 4; *Ho-1*: heme oxygenase 1; *Hsl*: hormone-sensitive lipase; *Lep*: leptin; *MnSod*: manganese superoxide dismutase; *Selenow*: selenoprotein W.

## Data Availability

The original contributions presented in this study are included in the article and Supplementary Materials. Further inquiries can be directed to the corresponding author.

## Supplementary Materials

The following supporting information can be downloaded at https://www.mdpi.com/article/10.3390/ijms27125345/s1.

## Abbreviations

BSU, Biological Services Unit; BW, Body Weight; CR, Calorie Restriction; DBP, Diastolic Blood Pressure; DIOs, Iodothyronine Deiodinases; *Dio2*, Type II Iodothyronine Deiodinase; *Fabp4*, Fatty Acid Binding Protein 4; *Fasn*, Fatty Acid Synthase; FGF1, Fibroblast Growth Factor 1; GPXs, Glutathione Peroxidases; *Gpx1*, Glutathione Peroxidase 1; *Gpx4*, Glutathione Peroxidase 4; HDL, High-Density Lipoprotein; HFD, High-Fat Diet; HFW, High-Fructose Water; HGF, Hepatocyte Growth Factor; *Ho-1*, Heme Oxygenase 1; *Hsl*, Hormone-Sensitive Lipase; ICP-MS, Inductively Coupled Plasma Mass Spectrometry; IGFBP-2, Insulin-like Growth Factor-Binding Protein 2; IGF-1, Insulin-like Growth Factor 1; LDL, Low-Density Lipoprotein; *Lep*, Leptin; MASLD, Metabolic-Dysfunction Steatotic Liver Disease; MetS, Metabolic Syndrome; *MnSOD*, Manganese Superoxide Dismutase; PAI-1, Plasminogen Activator Inhibitor-1; RBC, Red Blood Cells; SBP, Systolic Blood Pressure; sRAGE, Soluble Receptor for Advanced Glycation End-products; *Selenow*, Selenoprotein W; SD, Standard Diet; SD-ASe, Standard Diet with Adequate Selenium; Se, Selenium; TAC, Total Antioxidant Capacity; TRXRs, Thioredoxin Reductases; VLDL, Very Low-density Lipoprotein; vWAT, Visceral White Adipose Tissue; WD, Western Diet; WD-ASe, Western Diet with Adequate Selenium; WD-SOSe, Western Diet with Suboptimal Selenium.
