# Supplementary material for: Suboptimal Selenium Intake Produces Sex-Specific Alterations in Metabolic Profiles in Western Diet-Fed Obese Mice
Source: Int J Mol Sci. 2026 Jun 13;27(12):5345. doi: 10.3390/ijms27125345 (PMC13300374; doi:10.3390/ijms27125345)
Supplement: Supplementary file 1 [file ijms-27-05345-s001.zip › ijms-4310561-supplementary.pdf]

Supplementary Table 1. Plasma adipokines in male mice. Data is normalised to a reference protein and expressed as mean pixel density ± SEM.

Adipokine	SD-ASe	WD-ASe	WD-SOSe
Adiponectin	0.0809±0.0066	0.0866±0.0034	0.0873±0.0007
AgRP	0.0038±0.0005	0.0042±0.0001	0.0042±0.0001
ANGPT-L3	0.0676±0.0162	0.0563±0.0007	0.0511±0.0022
C Reactive Protein	0.0836±0.0079	0.0617±0.0011	0.0629±0.0027
DPPIV	0.0934±0.0067	0.0760±0.0066	0.0764±0.0031
Endocan	0.3082±0.0779	0.2569±0.0071	0.2373±0.0208
Fetuin A	0.0439±0.0022	0.0296±0.0073	0.0285±0.0091
FGF-1	0.0026±0.0003	0.0016±0.0002	0.0020±0.0004
FGF-21	0.0273±0.0211	0.0380±0.0011	0.0574±0.0102
HGF	0.0029±0.0006	0.0032±0.0015	0.0028±0.0013
ICAM-1	0.0761±0.0527	0.0681±0.0498	0.0714±0.0516
IGFBP-1	0.2767±0.0127	0.1893±0.0014	0.2580±0.0020
IGFBP-2	0.2044±0.0370	0.3112±0.0549	0.2985±0.0182
IGFBP-3	0.2608±0.0525	0.2070±0.0174	0.2187±0.0137
IGFBP-5	0.1813±0.0169	0.1327±0.0133	0.1417±0.0080
IGFBP-6	0.5987±0.0760	0.4313±0.1406	0.5048±0.0115
IGF-I	0.0166±0.0135	0.0333±0.0281	0.0311±0.0251
IGF-II	0.0065±0.0005	0.0077±0.0002	0.0063±0.0001
IL-6	0.0026±0.0004	0.0022±0.0006	0.0024±0.0008
IL-10	0.0035±0.0002	0.0022±0.0005	0.0023±0.0007
IL-11	0.0026±0.0006	0.0028±0.0011	0.0033±0.0014
Leptin	0.0090±0.0062	0.0286±0.0211	0.0345±0.0259
LIF	0.0038±0.0017	0.0044±0.0023	0.0041±0.0017
Lipocalin-2	0.1637±0.1532	0.1747±0.1684	0.1307±0.1229
MCP-1	0.0066±0.0003	0.0060±0.0002	0.0073±0.0001
M-CSF	0.0894±0.0066	0.0949±0.0005	0.0842±0.0036
Oncostatin M	0.0046±0.0009	0.0043±0.0002	0.0042±0.0003
PAI-1	0.0154±0.0011	0.0237±0.0007	0.0196±0.0054
Pentraxin 2	0.3082±0.0779	0.2569±0.0071	0.2373±0.0208
Pentraxin 3	0.0566±0.0095	0.0558±0.0067	0.0478±0.0001
Pref-1	0.0099±0.0009	0.0082±0.0013	0.0078±0.0001
RAGE	0.0189±0.0001	0.0049±0.0019	0.0076±0.0023
RANTES	0.0050±0.0008	0.0032±0.0011	0.0023±0.0010
RBP4	0.0149±0.0015	0.0122±0.0056	0.0138±0.0085
Resistin	0.1470±0.1223	0.1329±0.1207	0.1325±0.1199
TIMP-1	0.0035±0.0026	0.0028±0.0019	0.0036±0.0026
TNFα	0.0020±0.0001	0.0019±0.0001	0.0019±0.0002
VEGF	0.0107±0.0002	0.0083±0.0008	0.0078±0.0006

Supplementary Table 2. Plasma adipokines in female mice. Data is normalised to a reference protein and expressed as mean pixel density ± SEM.

Adipokine	SD-ASe	WD-ASe	WD-SOSe
Adiponectin	0.0534±0.0148	0.0590±0.0133	0.0555±0.0111
AgRP	0.0033±0.0004	0.0036±0.0004	0.0037±0.0008
ANGPT-L3	0.0625±0.0131	0.0554±0.0078	0.0466±0.0043
C Reactive Protein	0.0643±0.0111	0.0560±0.0099	0.0535±0.0113
DPPIV	0.0598±0.0080	0.0611±0.0060	0.0661±0.0082
Endocan	0.1853±0.0205	0.1951±0.0206	0.1997±0.0401
Fetuin A	0.0273±0.0044	0.0308±0.0031	0.0295±0.0013
FGF-1	0.0027±0.0004	0.0025±0.0002	0.0067±0.0014
FGF-21	0.0200±0.0024	0.0172±0.0001	0.0166±0.0027
HGF	0.0032±0.0003	0.0035±0.0003	0.0051±0.0006
ICAM-1	0.1049±0.0237	0.1096±0.0213	0.1181±0.0180
IGFBP-1	0.1713±0.0459	0.1263±0.0360	0.1617±0.0319
IGFBP-2	0.1859±0.0279	0.2822±0.0375	0.2973±0.0479
IGFBP-3	0.1697±0.0257	0.1482±0.0136	0.1699±0.0340
IGFBP-5	0.0971±0.0143	0.0953±0.0050	0.1166±0.0095
IGFBP-6	0.3836±0.0287	0.3923±0.0445	0.4604±0.0619
IGF-I	0.0542±0.0019	0.1577±0.0245	0.1381±0.0087
IGF-II	0.0056±0.0007	0.0079±0.0020	0.0074±0.0012
IL-6	0.0023±0.0002	0.0025±0.0001	0.0027±0.0001
IL-10	0.0029±0.0006	0.0027±0.0001	0.0033±0.0001
IL-11	0.0036±0.0006	0.0034±0.0001	0.0037±0.0001
Leptin	0.0104±0.0015	0.0566±0.0085	0.0896±0.0196
LIF	0.0049±0.0008	0.0042±0.0004	0.0060±0.0010
Lipocalin-2	0.2334±0.0466	0.2347±0.0404	0.3144±0.0621
MCP-1	0.0073±0.0007	0.0064±0.0001	0.0093±0.0011
M-CSF	0.0704±0.0203	0.0677±0.0175	0.0723±0.0179
Oncostatin M	0.0029±0.0002	0.0031±0.0003	0.0042±0.0005
PAI-1	0.0123±0.0001	0.0167±0.0003	0.0215±0.0005
Pentraxin 2	0.1853±0.0205	0.1951±0.0206	0.1997±0.0401
Pentraxin 3	0.0268±0.0003	0.0277±0.0026	0.0390±0.0067
Pref-1	0.0062±0.0001	0.0058±0.0003	0.0075±0.0003
RAGE	0.0195±0.0009	0.0089±0.0001	0.0210±0.0018
RANTES	0.0032±0.0001	0.0034±0.0001	0.0039±0.0001
RBP4	0.0213±0.0020	0.0226±0.0002	0.0227±0.0033
Resistin	0.3839±0.0015	0.3167±0.0325	0.4224±0.0052
TIMP-1	0.0075±0.0008	0.0091±0.0001	0.0112±0.0013
TNFα	0.0019±0.0003	0.0018±0.0004	0.0020±0.0001
VEGF	0.0068±0.0010	0.0050±0.0011	0.0070±0.0005
